# Loss of antioxidant function in donor human milk after holder pasteurization: a pilot study

**DOI:** 10.3389/fnut.2026.1761040

**Published:** 2026-03-16

**Authors:** Andrea Leong, Cristiane Mori, Christopher Pillidge, Harsharn Gill

**Affiliations:** School of Science, RMIT University, Melbourne, VIC, Australia

**Keywords:** antioxidant activity, donor human milk (DHM), holder pasteurization, Infant health, iron chelation and reduction

## Abstract

Human milk is considered the best food for infants. In certain instances, such as feeding premature babies, or babies with diseases requiring hospitalization, donor human milk (DHM) is used where mother's own milk is unavailable. DHM collection, treatment, frozen storage and distribution is managed by human milk banks, under strict Government regulations. Part of this process involves Holder pasteurization (HoP) of HM, which has been practiced for decades and is essential to ensure microbiological safety. However, HoP treatment also impacts on the nutritional and biological properties of HM. This study investigated the impact of HoP on the antioxidant properties of human milk (HM), focusing on its effects on free radical scavenging and metal ion chelation determined *in vitro* using standard biochemical assays on eight individual HM samples. The findings indicated that HoP significantly alters the antioxidant properties of HM. In particular, 1,1-diphenyl-2-picrylhydrazyl (DPPH) radical (↓18.4%, *P* < 0.001) and superoxide radical scavenging properties (↓ 9.1%, *P* < 0.05) and the potassium ferricyanide reducing capacity (↓ 17.4%, *P* < 0.01) of HM were all significantly reduced following HoP. In contrast, hydroxyl radical scavenging properties and iron chelation remained largely unaffected. Results further showed significant variability between individual HM samples, which may be attributed to maternal factors, genetic differences and varying lactation stages of the milk samples tested. Overall, these results underscore the need to develop new improved HM treatment protocols that maintain HM's nutritional and physiological properties during processing by milk banks, while at the same time ensuring equivalent microbiological safety.

## 1 Introduction

Human milk (HM) is recognized for its antioxidant properties, which are crucial for infant health. Many HM compounds play a key role in mitigating oxidative stress and preventing associated health conditions, such as necrotizing enterocolitis, retinopathy of prematurity, and bronchopulmonary dysplasia, which are considered oxygen radical associated diseases (1–3). These HM antioxidant components neutralize free radicals, thus

protecting sensitive biological systems from oxidative damage generated during metabolic processes and environmental exposure (4). Notable antioxidants in HM are enzymes, such as superoxide dismutase (SOD) and the enzymes of the glutathione (GSH) system comprising glutathione peroxidase (GPx) and reductase (5). Along with such enzymes, there are also non-enzymatic antioxidants present such as vitamin C, vitamin E, and glutathione (6).

In cases where mother's own milk isn't available, and in special cases such as premature birth, the next best option as recommended by the World Health Organization is to use donor human milk (DHM). DHM is managed and processed by registered milk banks, responsible for screening of donors and pasteurization, storage and distribution of the donated milk. Recent research into the effects of heat treatment, specifically Holder pasteurization (HoP), has shown different effects on the antioxidant properties of HM (7); see also review by Juncker et al. (8). HoP has been shown to notably reduce the glutathione and transferrin levels in milk, consequently reducing total antioxidant activity (9, 10). Despite this, some antioxidant components, such as SOD, have been observed to remain relatively stable after pasteurization (11).

Due to these conflicting results, further studies are necessary to understand the detailed effects of HoP on various antioxidant components. This is crucial for evaluating its overall impact on the nutritional quality of HM and ensuring that premature infants continue to receive optimal protection from oxidative damage. Thus, the research question guiding the study was: Does HoP significantly alter the antioxidant activity of DHM? This was based on the hypothesis that the antioxidant activity of DHM would be significantly reduced following HoP, due to the degradation of heat-sensitive bioactive constituents.

## 2 Materials and methods

### 2.1 Human milk samples

The HM sampling protocol used in this study was approved by the RMIT University Human Research Ethics Advisory Committee (Reference 2021-23195-43363). Eight individual HM samples from donors in the mature stage of lactation (in raw, unpasteurized state prior to freezing) were provided by Australian Red Cross Lifeblood, in accordance with the Australian National Statement on Ethical Conduct in Human Research. Selection criteria for donors, milk collection protocols, milk processing, handling and storage were based on established standards (12).

After receival HM samples were thawed slowly at 4 °C to preserve their integrity, then gently homogenized and divided into two portions. One portion remained unpasteurized (raw) while the other was heat-treated under Holder conditions (62.5 °C for 30 min in a water bath). The samples were aliquoted into 2 mL PP tubes and stored at −80 °C until further analysis.

### 2.2 Antioxidant assays

The antioxidant assays used in this study are described in detail by Leong et al. (13) and are included in the supplementary file. These assays are standard *in vitro* antioxidant assays, and are based on previously published methods: DPPH radical scavenging assay is based on the studies of Liu et al. and Zarban et al. (14, 15); the hydroxyl radical scavenging assay is based on (16); the superoxide radical scavenging assay is based on (17), and the Fe^2^^+^-chelating and potassium ferricyanide ([Fe(CN)6]3–) -reducing (FER) assays are both based on Liu et al. (18).

### 2.3 Statistical and data analysis

Antioxidant assays were performed on individual HM samples (*n* = 8) with technical triplicates. Technical replicates were averaged to obtain a single value per donor per condition prior to statistical analysis. The results for each experiment are presented both as individual and combined data showing the mean ± standard error (SE) for the grouped data. The differences between sample groups and controls were tested for significance by two-way ANOVA and, subsequently, Tukey's *post-hoc* test. Statistical analyses were done using GraphPad Prism version 7 for Windows GraphPad software. Statistical significance is defined as *P* < 0.05 (^*^), *P* < 0.01 (^**^) or *P* < 0.001 (^***^).

## 3 Results

### 3.1 Antioxidant properties

**Figure 1:**
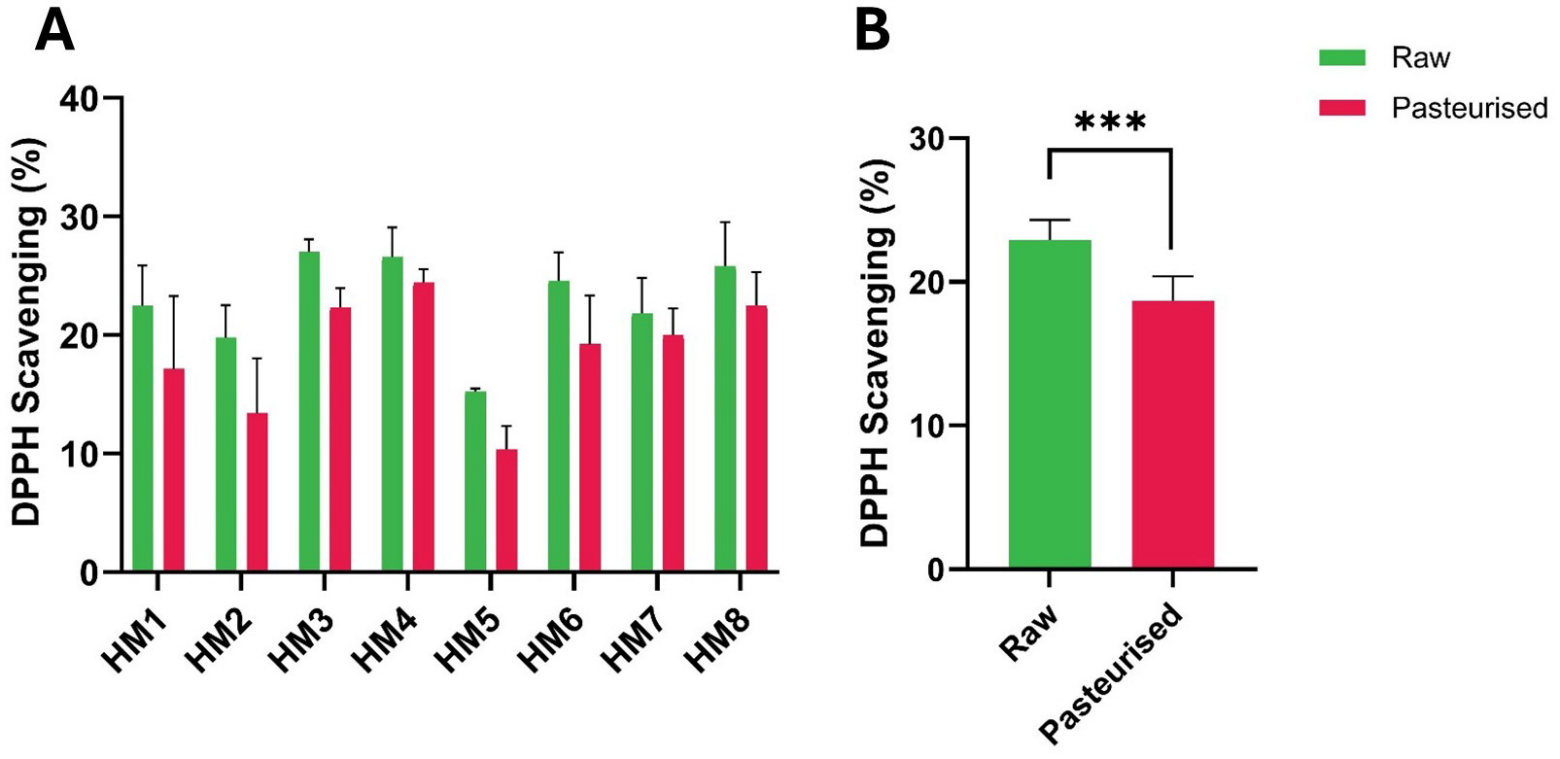
DPPH radical scavenging ability (%) of raw and Holder-pasteurized HM. **(A)** DPPH scavenging activity of individual HM donors; error bars represent the mean ± SE of three technical replicates **(B)** DPPH scavenging activity (mean ± SE) of raw and HoP-treated HM, grouped data (***, *P* < 0.001). The green bars represent raw milk, while the red bars represent pasteurized milk.

**Figure 2:**
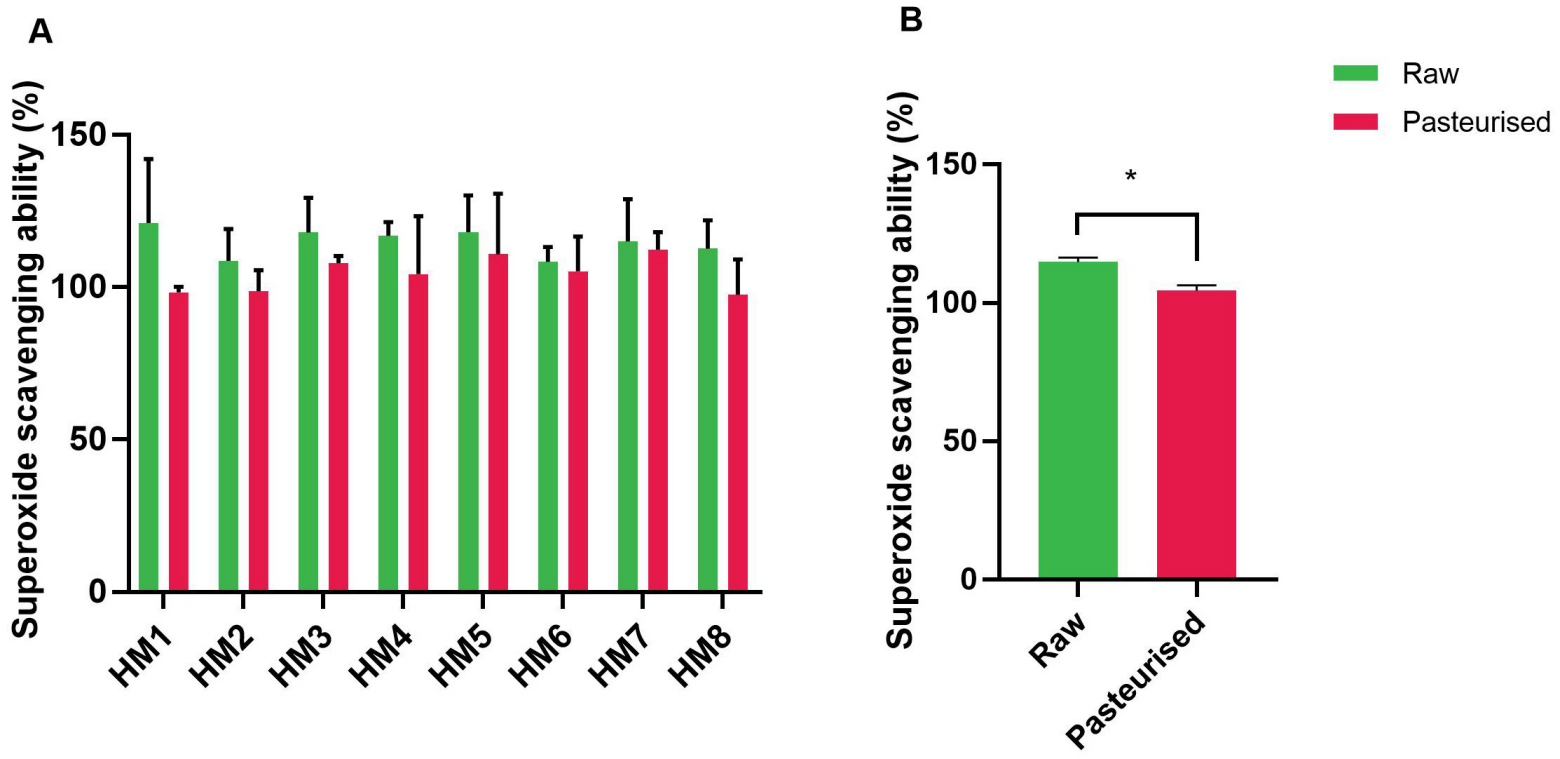
Superoxide scavenging ability (%) of raw and Holder-pasteurized HM. **(A)** Superoxide scavenging activity of individual HM donors; error bars represent the mean ± SE of three technical replicates; **(B)** Superoxide scavenging activity (mean ± SE) of raw and HoP-treated HM, grouped data (*, *P* < 0.05). The green bars represent raw milk, while the red bars represent pasteurized milk.

The free radical scavenging activities of HM before and after Holder pasteurization (HoP) were determined. DPPH radical scavenging activity varied markedly between donors (Figure 1A). However, following HoP, the activity decreased for all samples, with differences between the two treatment groups (raw vs pasteurized) being highly significant (*P* < 0.01; Figure 1B). Similarly, treatment of HM samples by HoP resulted in a decreased superoxide radical scavenging activity (Figure 2A). The average superoxide radical scavenging activity of HoP-treated HM (grouped data) was also significantly lower (*P* < 0.05) than that of the raw HM (Figure 2B). The average grouped values for DPPH scavenging activity for raw HM and HoP-treated HM were 22.91% and 18.68% respectively with a decrease of 18.4% (*P* < 0.0002) post-HoP. Similarly, the superoxide radical scavenging activity was reduced by 9.1% (*P* < 0.05) post HoP (Figure 2B).

**Figure 3:**
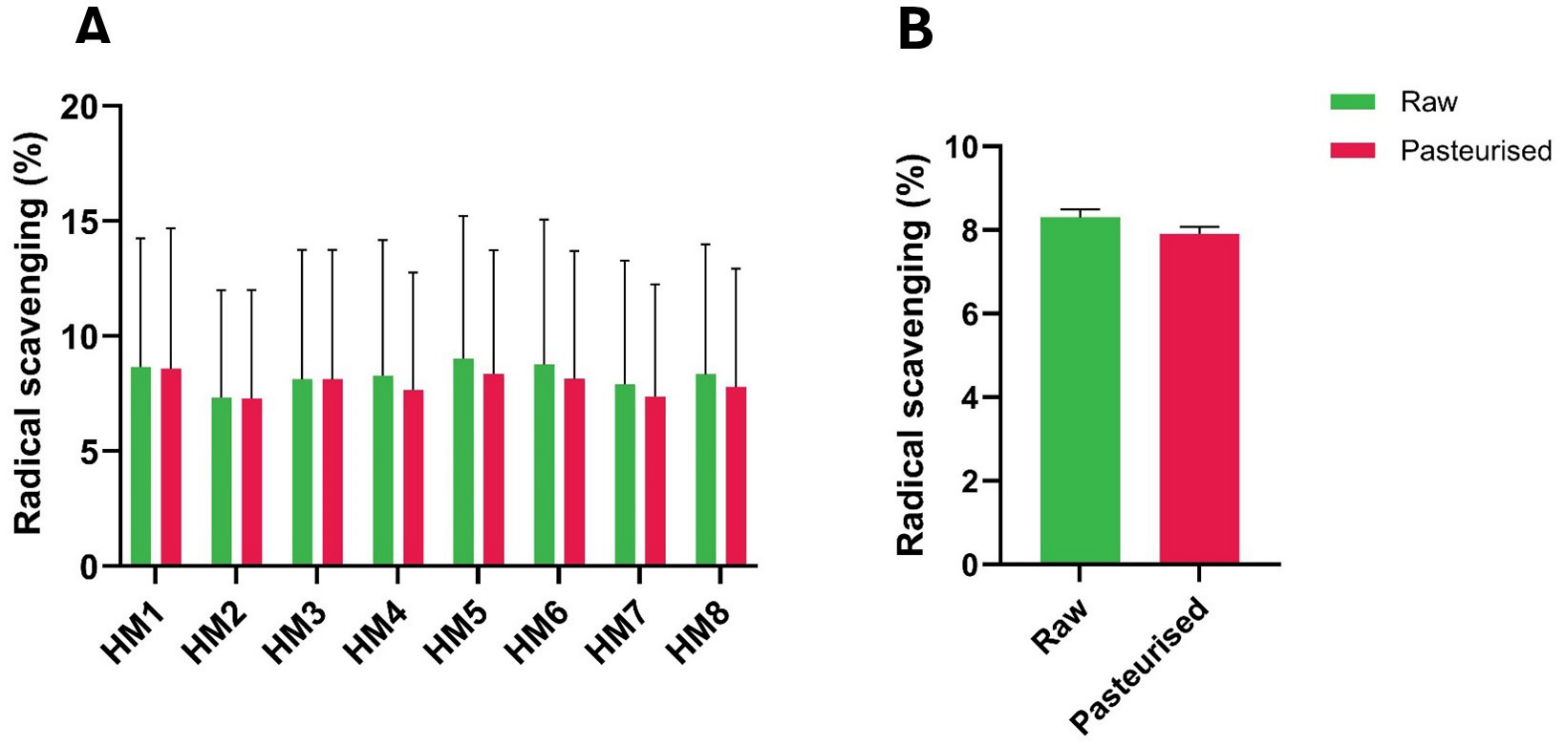
Hydroxyl radical scavenging ability (%) of raw and Holder-pasteurized HM. **(A)** Hydroxyl radical scavenging activity of individual HM donors; error bars represent the mean ± SE of three technical replicates; **(B)** Hydroxyl scavenging activity (mean ± SE) of raw and HoP-treated HM, grouped data. The green bars represent raw milk, while the red bars represent pasteurized milk.

In contrast to these results, the hydroxyl radical scavenging activity between raw HM and HoP-treated HM did not vary significantly (*P* > 0.05, Figure 3).

### 3.2 Iron-chelation and potassium ferricyanide reducing abilities

**Figure 4:**
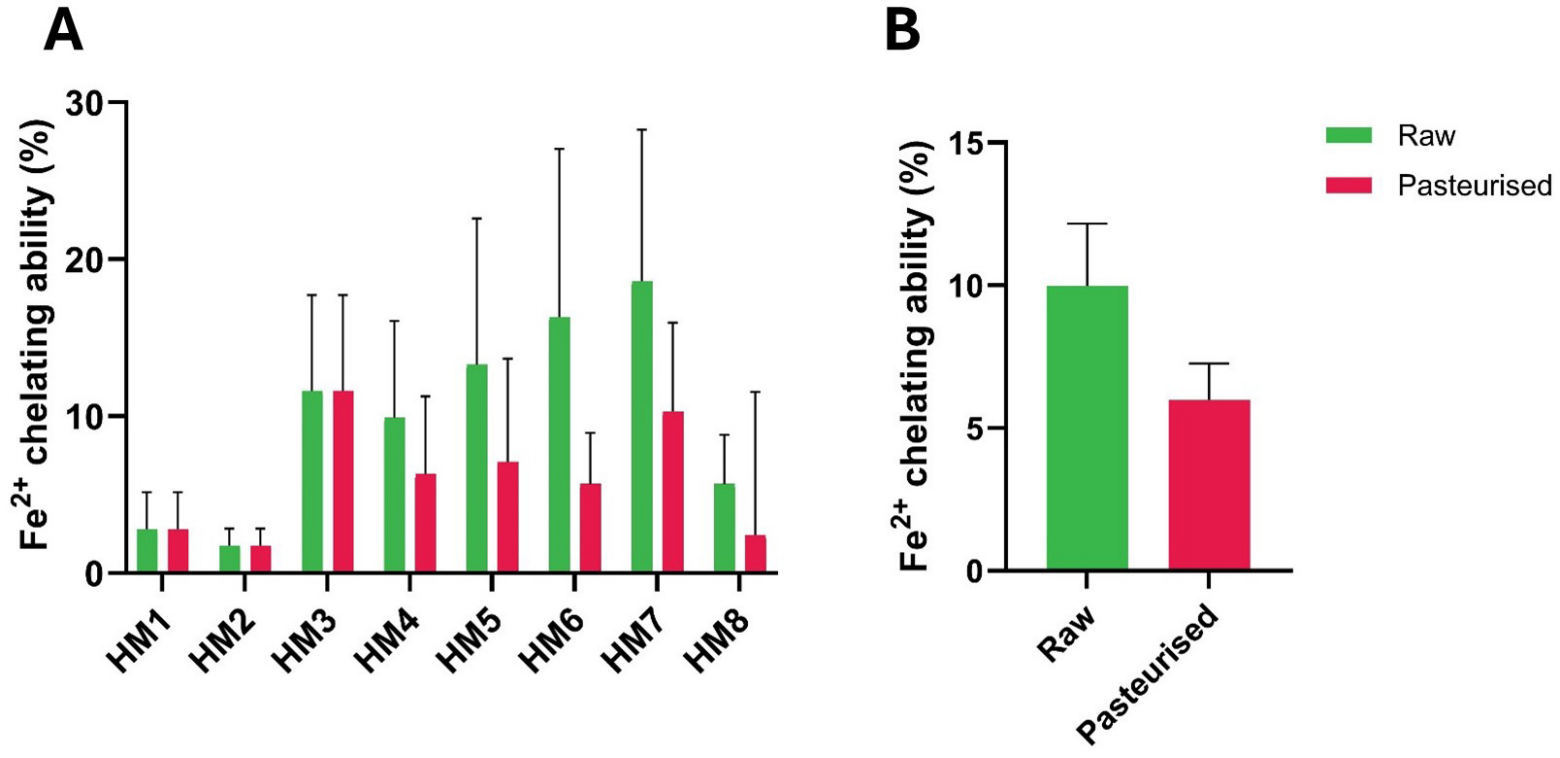
Fe^2+^ chelating ability (%) of raw and Holder-pasteurized HM. **(A)** Fe^+^ chelating activity of individual HM donors; error bars represent the mean ± SE of three technical replicates; **(B)** Fe^+^ chelating activity (mean ± SE) of raw and HoP-treated HM, grouped data. The green bars represent raw milk, while the red bars represent pasteurized milk.

**Figure 5:**
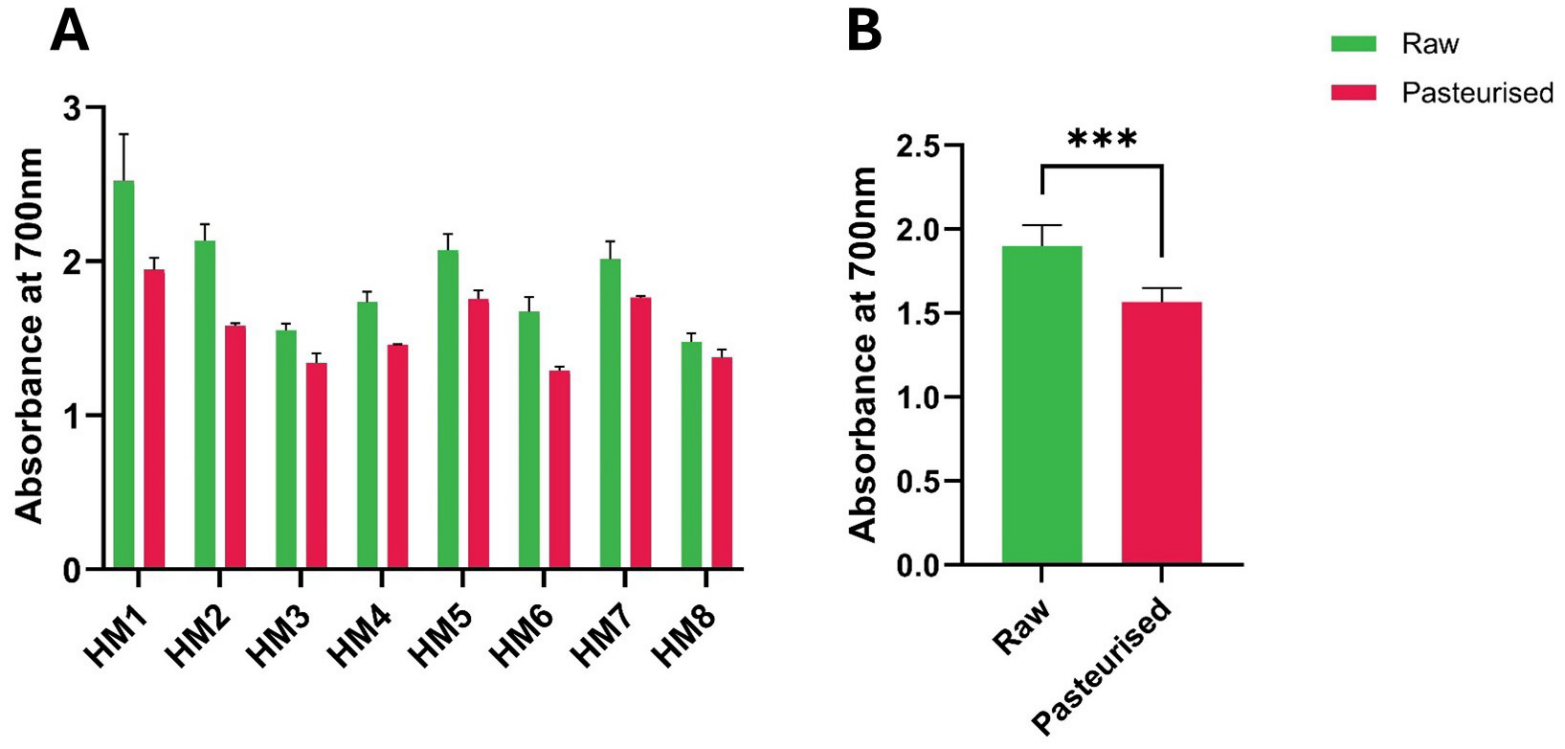
Potassium ferricyanide reduction measured by absorbance of raw and Holder-pasteurized HM. **(A)** Potassium ferricyanide reduction of individual HM donors; error bars represent the mean ± SE of three technical replicates; **(B)** Potassium ferricyanide reduction (mean ± SE) of raw and HoP-treated HM, grouped data (***, *P* < 0.001). The green bars represent raw milk, while the red bars represent pasteurized milk.

The Fe^2^^+^-chelating ability of samples is shown in Figure 4. Although decreases were observed at both the individual and group levels (Figure 4), the group differences were not statistically significant (Figure 4B). In contrast to this, the ferricyanide reducing ability of HoP-treated HM was demonstrably lower than that of raw HM for all eight individual samples (Figure 5A). Notwithstanding major inter-individual variability, the average potassium ferricyanide reducing activity of HoP-treated HM (grouped data) was significantly lower (17.4% less; *P* < 0.01) compared with that of raw HM (Figure 5B). Overall, these results show that HoP substantially inhibits the iron-reducing ability of HM.

## 4 Discussion

The results of this study showed a significant adverse impact of HoP on HM's antioxidant and iron-reducing activities, though not iron-chelating properties, determined by *in vitro* biochemical assays. Mean DPPH scavenging, superoxide radical scavenging and ferricyanide reduction activities of HoP-treated HM were all significantly lower than those of the raw HM. In contrast, the Fe^2^^+^-chelating ability showed no significant differences between raw and HoP-treated samples.

DPPH radical scavenging activity assays are commonly used to assess lipid oxidation. Upon reacting with an antioxidant capable of donating an electron or hydrogen atom, DPPH is reduced, resulting in a color change from violet to yellow. This change is accompanied by a decrease in absorbance, making the DPPH assay a simple and rapid method for measuring the free radical scavenging activity of antioxidants (19–22). The reduction in the average DPPH scavenging activity in HoP-treated samples (Figure 1) is consistent with earlier findings showing that HoP reduces the DPPH scavenging properties of HM (8, 23). This suggests that, despite the individual variations, there is a consistent overall reduction in antioxidant activity after pasteurization. Further studies are required to confirm the effect of HoP on antioxidants that prevent lipid oxidation, particularly since other findings indicate a decrease in fatty acids and an increase in methylene and ester functional groups, which are commonly associated with lipid oxidation. The hydroxyl radical antioxidant assay measures the ability of a substance to scavenge hydroxyl radicals, which are highly reactive oxygen species (ROS) that can cause oxidative damage in biological systems, particularly in vulnerable populations such as preterm infants (24, 25). In conditions of hyperoxia or reoxygenation, where mitochondrial production of superoxide anions and hydrogen peroxide increases, these ROS can react with ferrous iron to form hydroxyl radicals, which are capable of damaging lipids, proteins, and nucleic acids (24). Studies have suggested that hydroxyl radical scavenging properties in milk are due to specific components, including antioxidants such as vitamins, peptides, and polyphenols (26, 27). Our results indicate that HoP did not reduce milk's overall hydroxyl radical scavenging ability, corroborating the findings of other authors (28).

The superoxide anion assay measures the ability of a compound to scavenge superoxide radicals, which are ROS produced during aerobic metabolism. When present in excess, these ROS can contribute to oxidative damage in biological systems (29). In this assay, superoxide radicals are typically generated through the autoxidation of compounds such as pyrogallol in the presence of oxygen. The resulting superoxide anions are then detected using indicators, i.e. nitroblue tetrazolium, which turns blue upon reduction by superoxide radicals. The results of our study showed that HoP was associated with significant decrease in HM's superoxide anion scavenging properties. This reduction could be due to HoP inactivating some of superoxide scavenging compounds or enzymes, such as superoxide dismutase, catalase and glutathione peroxidase, which may reduce the milk's overall superoxide antioxidant capacity (28).

Superoxide dismutase, a key enzyme found in HM, plays an essential role in converting superoxide radicals into less harmful molecules, contributing to the milk's antioxidant defense system (30). Moreover, other proteins in HM, such as caseins and thioredoxin, have also been shown to effectively scavenge superoxide anions and other ROS (27, 31). Since some proteins, particularly whey proteins, lactoferrin, enzymes and immunoglobulins, are highly thermolabile, changes in their composition might have reduced superoxide scavenging properties.

The antioxidative strength of milk's components can also be measured by their ability to reduce ferric ion (Fe^3+^)-ligand complexes to ferrous (Fe^2+^) complexes. Transition metal ions promote lipid oxidation, leading to the formation of reactive peroxyl and alkoxyl radicals (32). Compounds with metal chelating properties can mitigate the oxidative process by binding to these ions and preventing them from catalyzing radical formation (33). In this study, there was a consistent decrease in the iron-chelating abilities of pasteurized milk for individual samples, however the combined difference between the two treatment groups was not statistically significant. Proteins such as casein, its byproducts, and lactoferrin have demonstrated strong iron-chelating properties (34, 35). Their iron-binding mechanisms have been extensively studied, showing how these proteins effectively sequester iron to reduce its pro-oxidant activity (36, 37). Lactoferrin, known for its iron-binding properties, can chelate metal ions that catalyze hydroxyl radical formation through Fenton reactions, thus indirectly reducing oxidative stress (38). This mechanism is crucial for preserving the antioxidative capacity of HM during processing.

The reduction of potassium ferricyanide in antioxidant assays is primarily facilitated by functional groups capable of donating electrons or hydrogen atoms (39). Key groups involved in this process include hydroxyl (-OH), amine (-NH_2_), thiol (-SH), and enol (-C(OH)=C-) groups. Through their electron-donating properties, these groups enable the conversion of potassium ferricyanide (containing a Fe3^+^ radical) into potassium ferrocyanide (Fe^2^^+^ radical) demonstrating the compound's reducing power. This reduction is a crucial indicator of antioxidative strength, as it reflects the substance's ability to neutralize reactive species. In milk, the components mostly responsible for this activity are proteins (with amine and thiol groups) and lipids (containing enol groups) (34, 35). Since the Fe molecule in the ferricyanide complex is in the +3-valence state, by inference the observed inhibition of its reduction by HoP could extend to other metal ions in an oxidized state.

The present results demonstrate that raw HM exhibits significantly higher reducing activity, as measured by the potassium ferricyanide assay, compared with pasteurized milk. Therefore, HoP significantly decreased milk's ability to reduce potassium ferricyanide, and by inference other metal ions, indicating a loss of antioxidant properties in the relevant functional groups. This effect was consistently observed across all individual samples, although the magnitude of reduction varied between mothers. When data were pooled, the difference remained highly significant, indicating that pasteurization negatively impacts the antioxidant properties of HM. The potassium ferricyanide assay reflects the presence of electron-donating antioxidants, including proteins such as lactoferrin, enzymes, peptides, and small molecules such as vitamins. The observed decline in absorbance at 700 nm after pasteurization is consistent with the known susceptibility of these bioactive components to heat-induced denaturation or degradation. These findings align with previous studies showing that HoP causes measurable decreases in antioxidant activity of human milk (10, 40, 41). Overall, HoP resulted in a decrease in HM antioxidant activity across all assays, with significant differences observed except for hydroxyl radical scavenging and iron-chelating assays where the reductions in activities were non-significant. Future research should explore alternative treatment methods for DHM, such as high-temperature short-time (HTST) pasteurization, retorting, high-pressure processing (HPP), and ultraviolet-C (UV-C) treatment. These approaches have demonstrated potential in preserving antioxidant properties more effectively than traditional Holder pasteurization, and they have been reviewed in detail elsewhere (9, 42). However, further studies are needed to confirm these findings and to assess their relative safety and impact on clinical outcomes in infants.

A large interindividual variation was observed possibly due to different lactation stages, dietary and lifestyle variations, health status and different genetic backgrounds of HM donors. In the present study, the lack of information on these variables limited our ability to investigate their potential effects. Future studies should aim to minimize these differences by utilizing a larger sample size and milk collection at defined stages of lactation.

The observed reduction in antioxidant activity of DHM, following HoP, is likely to have significant nutritional and clinical implications for pre-term, immunocompromised and otherwise medically compromised infants who are highly vulnerable to oxidative stress-related conditions (such as necrotizing enterocolitis (NEC), bronchopulmonary dysplasia (BPD), retinopathy of prematurity or damage to developing organs) due to immature defenses against oxidative stress (43–45). It may also impact recovery from infection, weaken immune protection and potentially impair growth and development (46–49). These conditions are major contributors to neonatal morbidity and mortality.

The study has several limitations. First, the small sample size, although adequate for detecting significant changes in antioxidant activity following HoP, limits generalization of these findings. Second, the lack of access to donor-specific information (such as stage of lactation, diet and other lifestyle factors) due to privacy reasons limited our ability to explain the possible effects of these variables. Third, the study did not include quantification of biologically active constituents (such as vitamins and proteins) and (fourth) direct assessment of health outcomes. These constraints and the fact that it is an *in vitro* study need to be considered when interpreting these findings and their clinical significance.

## 5 Conclusions

The study demonstrates that HoP reduces the antioxidant capacity of DHM, highlighting a tradeoff between microbial safety and preservation of the protective benefits of DHM for vulnerable neonates. Given that premature and newborn infants have immature physiological systems that are vulnerable to oxidative stress, the loss of antioxidant function may have clinical implications. These findings underscore the need for clinical studies to assess whether the reduced antioxidant capacity of DHM correlates with adverse health outcomes in recipient infants. Also, there is a need for ongoing risk-benefit analysis and exploration of alternative methods for better preserving the bioactivity of HM. Lastly, these findings underscore the importance of mother's own milk, when available, due to its superior retention of antioxidant bioactivity.

## Data Availability

The original contributions presented in the study are included in the article/supplementary material, further inquiries can be directed to the corresponding author.
